# Abnormal Pre-mRNA Splicing in Exonic Fabry Disease-Causing GLA Mutations

**DOI:** 10.3390/ijms232315261

**Published:** 2022-12-03

**Authors:** Franziska Alfen, Elena Putscher, Michael Hecker, Uwe Klaus Zettl, Andreas Hermann, Jan Lukas

**Affiliations:** 1 Translational Neurodegeneration Section “Albrecht-Kossel”, Department of Neurology, University Medical Center Rostock, 18147 Rostock, Germany; 2 Neuroimmunology Section, Department of Neurology, University Medical Center Rostock, University of Rostock, 18147 Rostock, Germany; 3 Center for Transdisciplinary Neurosciences Rostock (CTNR), University Medical Center Rostock, University of Rostock, 18147 Rostock, Germany; 4 Deutsches Zentrum für Neurodegenerative (DZNE) Rostock/Greifswald, 18147 Rostock, Germany

**Keywords:** missense mutation, alternative splicing, α-galactosidase A, exon skipping, intron inclusion, pharmacological chaperone

## Abstract

Fabry disease (FD) is a rare X-linked disease due to a multiverse of disrupting mutations within the *GLA* gene encoding lysosomal α-galactosidase A (AGAL). Absent AGAL activity causes the accumulation of complex glycosphingolipids inside of lysosomes in a variety of cell types and results in a progressive multisystem disease. Known disease-associated point mutations in protein-coding gene regions usually cause translational perturbations and result in premature chain termination, punctual amino acid sequence alterations or overall altered sequence alterations downstream of the mutation site. However, nucleotide exchanges at the border between introns and exons can affect splicing behavior and lead to abnormal pre-mRNA processing. Prediction with the Human Splicing Finder (HSF) revealed an indication of a significant change in splicing-relevant information for some known FD-associated *GLA* mutations. To experimentally determine the extent of the change, we made use of a minigene reporter assay and verified alternative splicing events for the exonic mutations c.194G>T and c.358C>G, which led to the usage of alternative donor splice sites at exon 1 and exon 2, respectively. In addition, the mutations c.548G>T and c.638A>T led to significant exon 4 skipping. We conclude that splicing phenotype analysis should be employed in the in vitro analysis of exonic *GLA* gene mutations, since abnormal splicing may result in a reduction of enzyme activity and alter the amenability for treatment with pharmacological chaperone (PC).

## 1. Introduction

Fabry disease (FD—OMIM 301500) is a rare X-linked lysosomal storage disease caused by the deficiency or absence of lysosomal α-galactosidase A (AGAL; EC 3.2.1.22). This defect leads to the inability of the lysosomes to catabolize complex glycosphingolipids. As a result, FD is a progressive multisystem disease [1]. Disease symptoms develop in both hemizygous males and heterozygous female carriers. FD usually affects males; however, due to X-chromosome inactivation, heterozygous females may exhibit disease manifestations presenting both mild and late symptoms as severe as phenotypes found in males [2,3]. Classical FD is caused by a complete loss of *GLA* gene (OMIM 300644) function due to the underlying mutation. It is defined by a complex multisystemic disorder with prominent features such as neuropathic pain, exercise intolerance, gastrointestinal abnormalities, progressive deterioration of renal and cardiac function, cerebrovascular involvement, and reduced life expectancy. A milder form of FD exists and is typically involved when residual AGAL activity is observed [4,5].

Often, a mutation occurs in only one family, such that the total number of mutations has already far exceeded the 2000 mark [6]. Most mutations are punctual nucleotide exchanges, insertions or deletions that alter the amino acid sequence in the AGAL enzyme or lead to premature chain terminations. A significant proportion of all alleles in the Asian population have an intronic change in the *GLA* gene, c.639+919G>A, resulting in an increase in physiological, alternatively spliced mRNA including a 57 nucleotide pseudoexon from intron 4 [7,8]. Patients with this mutation exhibit the cardiac variant of FD, suggesting that correctly spliced mRNA is still produced to generate a sufficient amount of normal-length active enzyme to prevent a classic FD phenotype. Another deep intronic mutation leading to activation of this pseudoexon and to unbalanced expression of this alternatively spliced *GLA* mRNA was also identified [9]. Other *GLA* gene mutations affecting pre-mRNA splicing have been investigated but are generally intronic and affect the canonical dinucleotides GT and AG for donor and acceptor sites or directly adjacent positions [10,11,12,13]. Deep intronic mutations are not detected by standard Sanger sequencing technology and require extended testing. Similarly, exonic mutations that affect the splicing process are difficult to predict, which may be a likely explanation for the fact that the predicted incorporation of an aberrant amino acid into the gene product in FD and other diseases is usually accepted as a disease-causing mechanism. However, a recently published study calculated a much higher rate of additional splicing effects of exonic mutations in general than currently estimated [14].

In previous studies, we investigated functional consequences of exonic point mutations leading to missense amino acid incorporations in the overexpression model [15,16]. These in vitro studies allowed for consideration of the damage to the altered AGAL enzyme forms based on this misincorporation. Due to the fact that they were based on heterologous expression and used cDNA vectors that did not contain introns, these studies were limited in that they could not illuminate possible splicing effects of the exonic mutations [17]. It is thus crucial to analyze the case that mutations affect a splice site, for example when ambiguous data are obtained by activity measurements in synthetic in vitro expression systems and patient-derived cells. We showed in the aforementioned previous publications that some of the mutations may have effects on pre-mRNA splicing. In these studies, we aimed to verify abnormal splicing using a minigene reporter assay on the most likely candidate mutations.

## 2. Results

### 2.1. In Silico Splicing Prediction

In our two previous publications, it was pointed out that some of the mutations studied were predicted to have evidence of aberrant splicing. These were c.194G>T (p.Ser65Ile), c.358C>G (p.Leu120Val), c.548G>T (p.Gly183Val), c.638A>T (p.Lys213Met), c.1025G>T (p.Arg342Leu), c.1115T>C (p.Leu372Pro) [15] and c.638A>G (p.Lys213Arg) [16]. We reexamined the mutations using the Human Splicing Finder (HSF) prediction program to reproduce the data obtained at that time since, in the meantime, an updated version of HSF was available. Table S1 summarizes the findings for the mutations at exon-intron junctions (A) and on putative cryptic splice sites (B). The findings for c.194C>T, c.358C>G and c.1115T>C still indicate an aberration in splicing and indicate the disruption or new formation of a donor splice site. The c.638 mutations affect position −2 proximal to the 5’ donor splice site of exon 4 and might putatively have an impact on the U1 snRNP-dependent initial step of spliceosome formation. Moreover, based on HSF predictions, changes in the cis-regulatory elements ESE (exonic splicing enhancer) and ESS (exonic splicing silencer) were identified for all mutations.

Enzyme activity and responsiveness to the pharmacological chaperone (PC) 1-deoxygalactonojirmimycin (DGJ, migalastat, Galafold^®^, Amicus Therapeutics, Cranbury, NJ, USA) were also redetermined and compared with previous study data (Table S2), because a slight adjustment of the measurement protocol was introduced after the initial measurement of the mutations [18], such that we wanted to ensure that the statements on the residual activity and the responsiveness of the mutations were verifiable. All mutations showed either significant residual activity or responsiveness toward DGJ comparable to the primary studies [15,16], meaning that p.Ser65Ile, p.Leu120Val and p.Gly183Val missense enzymes responded positively to DGJ, whereas p.Lys213Met and p.Lys213Arg did not. The high residual AGAL activity we measured for c.358C>G, c.638A>T and c.638A>G in the expression system may indicate that these mutations either cause mild FD symptoms or are even asymptomatic, because in patient cells ≥50% wild-type (WT) activity would be an unusually high activity for a symptomatic patient [19]. However, if other pathomechanisms such as abnormal pre-mRNA splicing intervene, activity in patient-derived cells would be further diminished, reinforcing the assumption that these variants are of pathological significance.

### 2.2. Corroboration of Splicing Abnormalities Due to GLA Exon 4 Mutations

**Figure 1:**
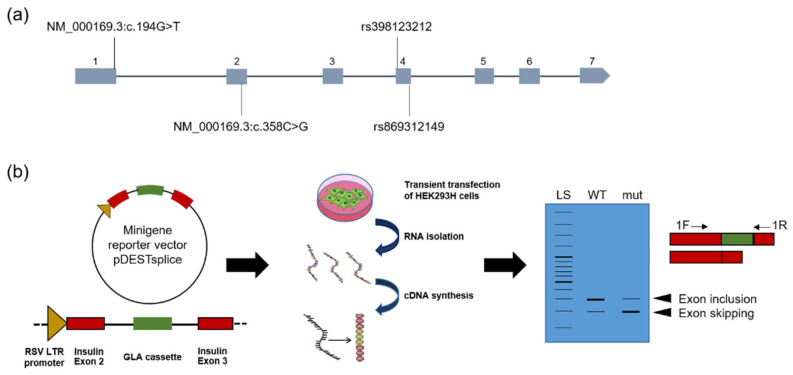
Location of the *GLA* gene mutations and general principle of the minigene assay. (**a**) Schematic view of the *GLA* gene locus (Xq22.1, X:101,397,803-101,407,925 according to GRCh38) with its 7 exons and the positions at which the exonic mutations analyzed by minigene assay are located. The mutations are indicated by their SNP ID. When no SNP ID was available, RefSeq # was used as the identifier. (**b**) The general principle of the Minigen assay. Minigene vectors of the type pDESTsplice were generated. These carry the *GLA* gene cassette consisting of the respective exon to be examined and about 400 base pairs of the adjacent intron regions. This minigene is inserted between exon 2 and 3 of the rat insulin gene under the control of a strong promoter. HEK293H cells are transiently transfected with these vectors. The cells are harvested, and total RNA is isolated 48 h post-transfection. The reverse transcribed cDNA is subjected to PCR using minigene-specific primers, and PCR products are analyzed using agarose gel electrophoresis.

A minigene assay was designed to validate the predicted effects on splicing. The intragenic location of the mutations is shown in Figure 1a. The mutations c.1025G>T and c.1115T>C are not indicated, because they were excluded from the minigene assay due to the fact that the missense enzymes did not show any residual activity in the in vitro system, and therefore already showed the most severe biochemical phenotype possible. There was no response to PC either [15]. Moreover, these mutations are located in the last *GLA* exon 7, and we deemed that analysis in the minigene assay was not meaningful. pDESTsplice was used for the generation of the reporter constructs. To this end, either the wild-type or mutant *GLA* exon and adjacent introns were inserted as previously suggested [20] (Figure 1b). After transfection of HEK293H cells, total RNA was isolated, cDNA was synthesized and splicing analysis of the minigene was carried out using PCR.

**Figure 2:**
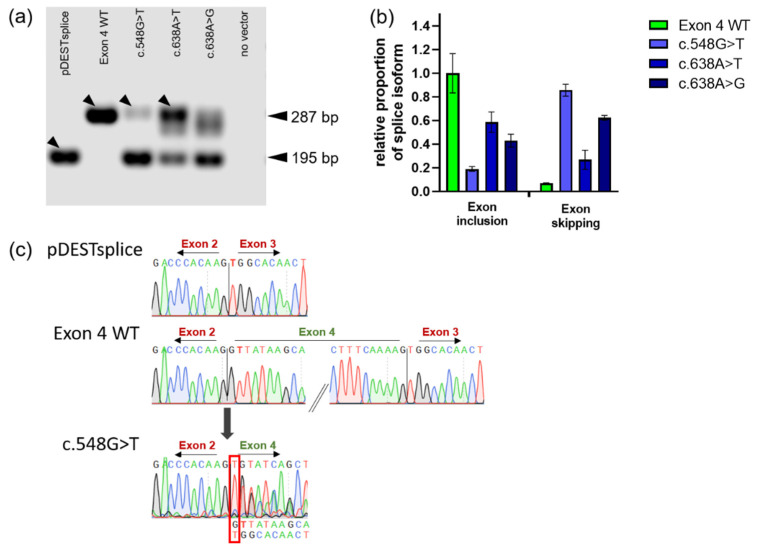
Splicing analysis of *GLA* exon 4 mutations using minigene reporter assay demonstrates *GLA* exon 4 skipping. (**a**) HEK293H cells were transiently transfected with *GLA* minigenes of wild-type exon 4 or the respective point mutation. Total RNA was isolated 48 h post-transfection, and cDNA was synthesized using random hexamer oligonucleotides to prime the reaction. Splicing products were then analyzed using rat insulin exon specific primers. The lower band represents splicing forms that do not contain *GLA* exon 4. The upper band indicates the correct splicing form containing exon 4. The arrows indicate the bands that were isolated and sequence-analyzed using Sanger technology. (**b**) Quantification of the band signals representing the relative proportion of the splicing isoforms. First, all signals were set in the ratio of the band for 18S ribosomal RNA. To determine exon inclusion, this ratio was normalized to 1 for the WT exon 4 minigene. For exon skipping, all results were related to the lower band result obtained for pDESTsplice. (**c**) Sanger sequencing of the indicated bands from (**a**). The sequence sections show the emerged exon/exon junctions at the respective mutated 3’ acceptor and 5’ donor splice sites. For c.548G>T the human *GLA* wild-type exon 4 and rat insulin exon 3 sequences are indicated below the chromatogram. The red box shows the cDNA position c.548. Rat insulin exons are typed in red, the *GLA* exon is typed in green.

The *GLA* exon 4 construct harboring wild-type sequence (Exon 4 WT) was used as a positive control for the mutations c.548G>T, c.638A>T and c.638A>G. The pDESTsplice-Exon 4 WT vector showed the expected normal splicing behavior including *GLA* exon 4 in between rat insulin exons 2 and 3 as indicated by a band at 287 bp (Figure 2a). Transfection of the pDESTsplice empty vector served as the negative control, and a fragment representing only the rat insulin exons was detected by the length of the expected 195 bp. The mutations c.548G>T (corrupted 3’ acceptor splice site), and c.638A>T and c.638A>G (corrupted 5’ donor splice site) showed both the upper and lower bands, suggesting aberrant pre-mRNA splicing in the form of skipping of exon 4. Quantitative analysis of the obtained signal revealed that c.548G>T retained less than 20% of the normally spliced RNA. Therefore, exon skipping seems to be the more common case than exon inclusion (Figure 2b). Although, in comparison, a stronger signal of the higher molecular weight band is perceptible for c.638A>T and c.638A>G, indicating a greater proportion of normally spliced RNA, the “smear” at approximately 287 bp indicating that more splice forms may very likely have been formed.

We examined the tagged bands (Figure 2a) in more detail by having them excised from the gel and subjected to sequencing analysis. The band obtained with pDESTsplice vector without a *GLA* cassette showed the expected splice product with the junction between rat insulin exons 2 and 3 (Figure 2c). Sequencing of the band obtained from the construct harboring *GLA* Exon 4 WT showed normal splicing. The c.548G>T-associated fragment showed superimposed DNA signals in the Sanger sequencing. The superimposed signal showed identity to exon 3 of rat insulin. Accordingly, isolating the band representing the correct splicing product harboring the guanine to thymine substitution proved to be difficult. The chromatogram for mutation c.638A>T was not evaluable due to even stronger superimposed traces and is therefore not included in Figure 2c. We suggest that our failure to isolate the PCR product related to the normal splicing form was due to the presence of further abnormal splicing events involved that utilized alternative splice sites in the absence of a strong canonical splicing signal.

The guanine at position c.548 of the *GLA* gene represents the first nucleotide of exon 4 and likely has an impact on splicing site recognition. Other mutations at this position are also related to FD, c.548G>A (p.Gly183Asp) and c.548G>C (p.Gly183Ala) [21,22], and were analyzed as well. Abnormal exon 4 skipping was also observed in these two mutations (Figure S1a), which have reported in vitro enzyme activity of 0.7 and 22.4% WT, respectively, and are among the strong PC responders [23]. As a mutation with confident splicing damage, the mutation c.639+1G>C at the canonical donor splice site was also included. As expected, this variant showed mainly exon 4 skipping (Figure S1b). The detected band appeared to represent an alternative splice form, possibly using the putative donor site at c.609/c.610. The expected fragment length would be 256 bp, which matched the calculated fragment length of 259 bp fairly well. However, we did not investigate this further.

**Figure 3:**
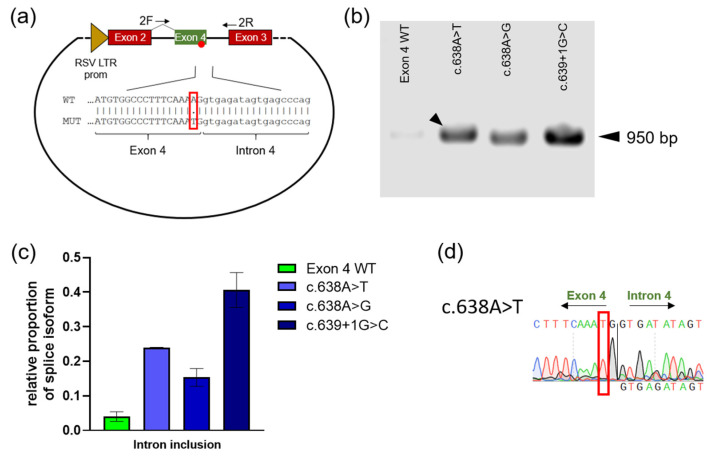
Mutations at the 5’ donor splice site of exon 4 lead to intron inclusion in the minigene reporter assay. (**a**) Schematic representation of the minigene vector and PCR primers used to detect intron 4 inclusion. Primer 2F spans rat insulin exon 2 and human *GLA* exon 4 such that the primer cannot bind the reporter vector product without insert; primer 2R overlaps the 3’ intron region adjacent to rat insulin exon 3. The red boxes indicate rat insulin exons, the green box indicates human *GLA* exon. The EMBOSS Needle Pairwise Alignment Tool [24] was used to visualize the exon–intron sequence. (**b**) The result of PCR detects the band at approximately 950 bp only for the exon 4 mutations used and the splice site-disrupting variant c.639+1G>C, but not for the wild type. (**c**) Quantification of (**b**). For visualization, the band signals obtained were related to the signals for 18S ribosomal RNA. (**d**) Sanger sequencing of the c.638A>T band (see arrow in (**b**)). The red boxes in (**a**,**d**) show the cDNA position c.638.

Furthermore, we explored whether the c.638A>T, c.638A>G, and c.639+1G>C constructs can give rise to splice variants that include the adjacent intron, in addition to the observed splicing products that lack exon 4. To this end, specific exon/exon and exon/intron spanning primers were designed (Figure 3a, Table S3), which only give a PCR product if intron 4 is not properly excised. All *GLA* construct variants except for the wild type showed the corresponding band at 950 bp (Figure 3b). Quantitative analysis showed that the classic c.639+1G>C mutation generated the strongest intron retention signal (Figure 3c). The band of c.638A>T was isolated and subjected to sequence analysis to confirm that the expected exon–intron junction had emerged (Figure 3d).

In conclusion, it can be deducted that the exon 4 mutations under investigation lead to reduced amounts of correctly spliced mRNA and thus to a reduced synthesis of functional AGAL.

### 2.3. Utilization of Alternative Splicing Sites as a Result of Mutations in Exons 1 and 2

**Figure 4:**
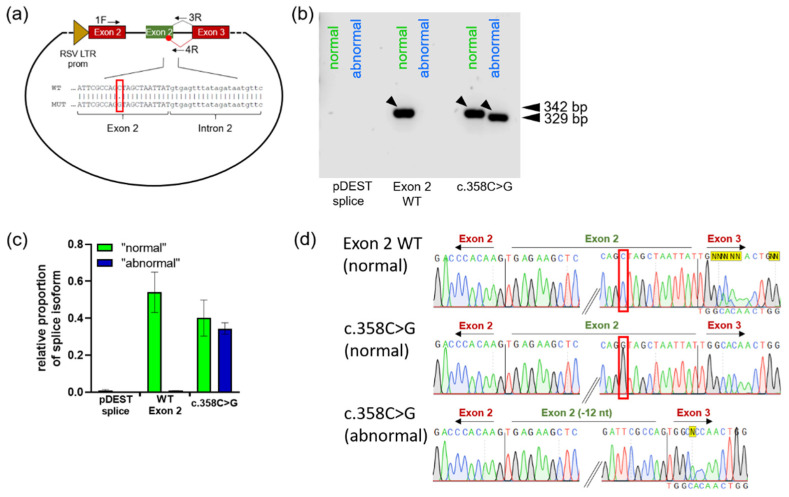
c.358C>G activates a cryptic 5’ donor splice site within exon 2. (**a**) Schematic representation of the minigene vector and PCR primers used to detect abnormal utilization of the intraexonic cryptic splicing site of exon 2. The 1F primer represents the standard pDESTsplice vector primer specific for rat insulin exon 2. The two reverse primers use either the first regular 5’ nucleotides of exon 2 or the alternative splice junction 12 nucleotides upstream. The red boxes indicate rat insulin exons, the green box indicates human *GLA* exon. (**b**) Agarose gel band labeled “normal” shows the correctly spliced RNA resulting from both PCR (primer 1F/3R) with the exon 2 WT construct transfected cells and the c.358C>G mutation. The band labeled “abnormal” indicates a fragment shorter by 12 nucleotides after altered pre-mRNA splicing. (**c**) Quantification of the band signals from (**b**). Shown here is the ratio of the respective band signal and the band signal for 18S ribosomal RNA. (**d**) Sanger sequencing of PCR fragments marked with arrows in (**b**). The red boxes show the cDNA position c.358. In case of an unclear Sanger result, the expected WT sequence was inserted below the chromatogram.

Although the exon 2 base substitution at position c.358 leads to the replacement of a highly conserved amino acid, the enzyme variant was measured with 42.0 to 66.8% WT activity in different expression systems involving either COS-1 or HEK293 cells [23,25]. In evident contrast to this high residual activity, there was an activity measured at less than 5% of the normal value in isolated patient leukocytes [25]. However, because this mutation was found in neonatal testing and no parental data were published, this variant was considered likely to cause late-onset FD. HSF prediction suggests a possible change in splicing due to activation of a cryptic 5’ donor splice site. By the use of exon–exon- and intron–exon-specific primers (Figure 4a, Table S3), the visualization of a shortened alternatively spliced RNA form was achieved (Figure 4b). The band signal of the normally spliced form was also slightly reduced (Figure 4c). Sequence analysis identified an RNA shortened by 12 nucleotides using the predicted newly formed donor splice site (Figure 4d). This splicing phenotype may explain the discrepancy between the ex vivo and in vitro enzyme activity measurements and implies higher-order damage that may be caused by c.358C>G.

**Figure 5:**
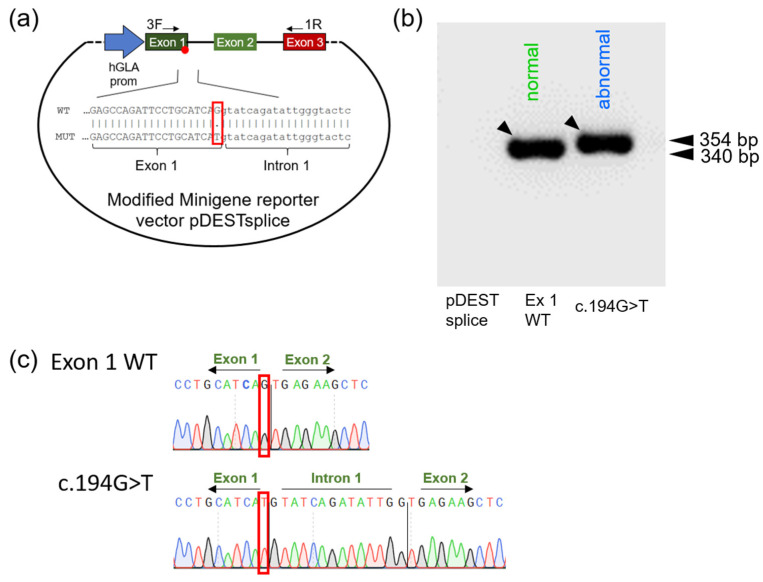
c.194G>T disrupts the canonical exon 1 AG-GT donor splice site and leads to abnormal splicing. (**a**) Schematic representation of the modified vector used for the minigene reporter assay. The strong RSV LTR promoter and rat insulin exon 2 were removed and replaced instead with approximately 1000 bp of human *GLA* core promoter and *GLA* exon 1. Human *GLA* exons are shown in green, the rat insulin exon in red. (**b**) PCR performed with primer pair 3F/1R shows different running behavior of the obtained PCR fragments for exon 1 WT and c.194G>T. The arrows indicate the bands that were isolated and sequence-analyzed using Sanger technology. (**c**) Sanger sequenc-ing of PCR fragments marked with arrows in (**b**). Sequence analysis of the bands indicates the presence of an RNA molecule elongated by exactly 14 nucleotides, resulting from the use of an alternative splicing signal within intron 1. The red boxes show the cDNA position c.194.

It has been suggested that the exon 1 mutation c.194G>T leads to amino acid substitution from serine to isoleucine in the AGAL enzyme [15]. The c.194G>C mutation leading to serine substitution by threonine was also treated as a missense mutation in previous studies [26,27]. To investigate whether c.194G>T causes a splicing defect, the pDESTsplice reporter plasmid was modified by replacing both the rat insulin exon 2 and the strong Rous sarcoma virus long terminal repeat promoter under whose control expression occurs with a *GLA* cassette consisting of the human *GLA* core promoter as well as *GLA* exon 1 and exon 2 and portions of adjacent introns (Figure 5a). The primers used to analyze expressed RNA amplify a fragment 339 bp in length in the case of normal pre-mRNA splicing. Cells transfected with the exon 1 WT construct expressed the expected normal length fragment. When cells were transfected with the variant c.194G>T minigene, the PCR product was several nucleotides longer (Figure 5b). Sequencing of the isolated DNA fragments indicated that upon loss of the guanine at the last position of exon 1, an alternative donor splice site in intron 1 involving the nucleotides GG-GT (c.194+13-16) was used (Figure 5c).

## 3. Discussion

Fabry disease is a rare monogenic X-linked disease of glycosphingolipid metabolism. It is thought that a mutation leads to the onset of the disease by reducing AGAL enzyme activity below a critical threshold through substrate accumulation of globotriaosylceramide and globotriaosylsphingosine [28,29,30]. The disease also affects women mostly with milder courses. For prognostic and therapeutic reasons, the consideration of pharmacogenetics using molecular biology methodology and quantitative statistics to investigate the defective mechanism of a mutation becomes increasingly important. Hence, in vitro enzyme activity in heterologous expression systems has now been incorporated into FD management as part of the guidelines and consensus recommendations [5]. However, the in vitro activity measurement also has drawbacks, since on the one hand, only exonic mutations can be investigated, and on the other hand, even in the case of exonic mutations, only the theoretical missense enzyme form can be analyzed. A good laboratory practice (GLP)-validated enzyme activity assay was found to be acceptable in the DGJ approval process for predicting responsiveness or amenability of a *GLA* mutation to pharmacological chaperone therapy (PCT) [31].

Several reports in the past have shown that mutations can exist in the *GLA* gene at intron/exon boundaries as well as in deep intronic mutations that can exert devastating effects on gene function by altering splicing behavior and thus ultimately lead to FD [7,9,10,12,13]. In addition, there is an understanding that exonic mutations that rather contribute to defective splicing might be misclassified as missense mutations [32]. Nevertheless, no systematic analysis of such exonic putative splicing mutations has been conducted. Previous reports examined patients with mutations suspected of having an abnormal pre-mRNA splicing phenotype in the *GLA* gene followed by molecular studies. In the present study, the reverse approach was taken. Different *GLA* gene mutations were systematically analyzed for splicing abnormalities.

The mutations c.194G>T, c.358C>G, c.548G>A, c.548G>C, c.548G>T, c.638A>G and c.638A>T were examined by a minigene reporter assay, which can identify abnormal pre-mRNA splicing. Deviations from the normal splicing pattern were found for all mutations examined. For the exon 4 mutations, varying levels of exon 4 skipping and the possibility of intron retention were observed. For the exon 1 mutation c.194G>T, evidence was found for the emergence of an mRNA form that is 14 nucleotides longer than the wild type by use of an intra-intronic alternative splice site. The exon 2 mutation c.358C>G revealed, in addition to normal splicing, a splicing form shortened by the 12 terminal 3’-nucleotides from exon 2. However, as with c.548G>T, we succeeded in isolating a PCR band corresponding to correctly spliced missense RNA also for c.358C>G (Figure 2c and Figure 4d). The results of all variants examined suggested that larger proportions of the mRNA corresponded to alternatively spliced forms that cannot give rise to a functional enzyme.

These findings have a significant impact on FD management. In 2016, PCT was introduced for the treatment of FD [33]. PCT is based on the concept that missense mutations often cause protein misfolding, giving rise to abnormal conformations that can be corrected by small-molecule ligands that can interact with the mutant enzyme to emend its conformation, increase stability and lead to the recovery of corrective levels of enzyme in the lysosomes [34]. Since this approach is only viable for a fraction of *GLA* gene mutations [23,35], the mechanism responsible for the loss of function becomes of crucial relevance. Since a splicing defect may reduce the amount of folding-deficient missense enzyme amenable to PCT, splicing studies should be considered prior to a treatment decision. At least for mutations for which in vitro enzyme activity is not compliant with clinical and paraclinical data such as ex vivo enzyme activity in leukocytes or plasma biomarkers, splicing abnormalities could be a very likely risk factor for insufficient PCT efficacy. There may be strong discrepancies between ex vivo AGAL activity in patient cells and in vitro measurement. As is clear in the case of the c.358C>G mutation, the value of less than 5% of normal activity measured in leukocytes and the in vitro measurement of p.Leu120Val from overexpressing cells [25] is probably not solely explained by the difference in experimental approach, but by abnormal splicing processes in patient cells that evade detection in the expression system. Unfortunately, no accurate monitoring of data from different cell systems currently exists. However, a comparison of several studies found these similar discrepancies of lower ex vivo activity for the c.540G>T (p.Leu180Phe), c.644A>G (p.Asn215Ser) and c.758T>C (p.Ile253Thr) mutations in additional individual cases [17]. In our re-examination using HSF for computational splicing prediction, some mutations had little effect, e.g., c.548G>T. This raises the question of whether abnormal splicing may also play a role in mutations that were not suspected of contributing to splicing defects and that were previously classified as GVUS or benign polymorphisms by high in vitro activity. Such candidate mutations include p.Leu180Phe and p.Ile253Thr, which are considered clinically mild but have higher enzyme activity in the expression system than in patient cells, with 32.4% and 73.0% residual activity in HEK293H [16]. Moreover, both missense enzyme variants are highly responsive to PC treatment in vitro.

The present study provides evidence of an aberrant pre-mRNA splicing in that case of the *GLA* mutations p.Gly183Ala and p.Gly183Asp as well as p.Lys213Met and p.Lys213Arg, which are currently classified as amenable to DGJ [6]. While evidence of aberrant splicing in cells of patients carrying these mutations is pending, increased attention to the treatment of these patients with the chaperone is warranted. The example of the putative splice-altering variant c.194G>T (p.Ser65Ile) shows that patients with a mutation biochemically classified as a responsive variant failed to clinically benefit from the treatment [23]. Therefore, this mutation is listed as non-amenable despite biochemical responsiveness in the GLP-HEK293 cell system [6]. The mutation was assigned the affix “putative splicing site” in the official Galafold Amenability Table. Our study shows further evidence for this. p.Leu120Val and p.Gly183Val were annotated as non-responsive in contrast to our own data (Table S2). The assessments of the p.Lys213 mutations also contrast between the official GLP-validated assay and our data [35]. Several factors presumably play a role: on the one hand, the different assay conditions and on the other hand, different criteria for the evaluation of amenability. Nevertheless, a good agreement of 87.1% in the amenability classification was observed between both studies (155/178) [35]. In the present study, however, the rate of congruent assessment of the seven missense mutations examined in the minigene assay was only 42.9% (3/7). However, the results shown for all seven mutations examined in the minigene assay suggest abnormal pre-mRNA splicing. In a recent study, antisense RNAs were found to efficiently correct deep intronic splice-modifying mutations in the *GLA* gene [36]. We speculate that the mutations presented here may benefit from the use of such mutation-specific antisense RNAs to enhance exon inclusion as in the case of exon 4 mutations and to favor the use of the constitutive acceptor/donor splice site as in the case of exon 1 and exon 2 mutations.

## 4. Materials and Methods

### 4.1. Splicing Prediction

The predicted impact on splicing signals was investigated using the Mutation Analysis Tool from HSF version 3.1 [37].

### 4.2. Cell Culture

The HEK293H cells were maintained in high glucose (4.5 g/L) Dulbecco’s Modified Eagle Medium (DMEM) supplemented with 10% FBS and 1% penicillin/streptomycin in a water-jacket incubator at 37 °C under a 5% CO_2_ atmosphere. The cells were subcultured at a density of 80–90%.

### 4.3. AGAL Enzyme Activity Measurement

In vitro enzyme activity measurement was performed as previously described [18]. In brief, HEK293H cells were transfected with the plasmid vectors containing the appropriate GLA variant cDNA using Lipofectamine 2000. Cells were washed with PBS 48 h post-plasmid transfection and harvested in ultrapure water and homogenized by freezing and thawing. After centrifugation at 10,000× *g* at room temperature, the protein concentration of the cleared lysates was determined using the BCA method. Equal amounts of total protein were mixed with the synthetic fluorigenic substrate 4-methylumbelliferyl-α-D-galactopyranoside, and the reaction was incubated for 1 h before the release of 4-methylumbelliferone was measured in a Spark multiplate reader (Tecan, Männedorf, Switzerland) at 360 and 465 nm (excitation/emission). Absolute enzyme activity data were corrected for endogenous enzyme activity of the HEK293H cells. To evaluate endogenous enzyme activity, the cells were transfected with an empty vector.

### 4.4. Construction of the Minigen Reporter Vectors

The minigene sequences were designed according to known protocols [20]. Customized synthesis of minigenes for the genomic fragments containing GLA exons 2 and 4 included in the donor vector pDONR221 were purchased from BioCat GmbH (Heidelberg, Germany). To produce the pDESTsplice reporter minigenes, Gateway™ cloning was performed using LR Clonase™ II (Thermo Fisher, Braunschweig, Germany) according to the manufacturer’s instructions. Briefly, the LR reaction consisting of entry clone (pDONR221 + GLA cassette) and destination vector (pDESTsplice empty vector, Addgene plasmid #32484) and the LR Clonase Enzyme blend were mixed and incubated at 25 °C for one hour. After completion of the reaction by addition of Proteinase K, the mixture was used to transform the F’-episome positive, T1 phage-resistant bacterial strain One Shot™ OmniMAX™ 2 (Thermo Fisher, Braunschweig, Germany). The next day, colonies were picked and correct clones were identified by restriction analysis and Sanger sequence analysis (MWG Eurofins, Ebersberg, Germany). The construction of the pDESTsplice GLA exon 1 minigene followed a two-step cloning process. First, using specific primers (Table S3), a 1270 bp fragment was amplified from human leukocyte genomic DNA containing the GLA promoter, exon 1, and part of intron 1. The primers inserted cleavage sites for the restriction endonucleases HindIII and BamHI, which were required for cloning into pDESTsplice between these cleavage sites. Digestion of pDESTsplice conveniently removed rat insulin 2 exon 2 and the RSV LTR promoter region. Successful ligation of the intermediate and transformation of E. coli strain XL10 Gold (Agilent Technologies, Santa Clara, CA, USA) was confirmed by restriction analysis and Sanger sequencing. The second PCR amplified another part of GLA intron 1, exon 2 and intron 2 and functionalized the resulting 775 bp DNA fragment again by restriction endonuclease cleavage sites for BamHI and NotI. The fragment was inserted into the corresponding unique cleavage sites in the pDESTsplice vector. Subsequently, mutagenesis PCR was performed using the quikchange site-directed mutagenesis kit according to the manufacturer’s instructions to obtain variant c.194G>T.

### 4.5. Isoform-Specific PCR Assay

First, 1.5 × 10^5^ HEK293H cells were seeded in each cavity of a 24-well plate one day prior to transfection with the pDESTsplice reporter constructs. Cells were transfected using Lipofectamine LTX Reagent according to the manufacturer’s instructions. After 4 h, a medium change was performed. After a total of 48 h post-transfection in culture, cells were washed with PBS, dissolved in RNA Lysis Buffer, and RNA was isolated using the associated Quick-RNA Miniprep Kit (Zymo Research, Freiburg i. Breisgau, Germany). RNA concentration was determined in a Spark Microplate Reader (Tecan, Männedorf, Switzerland) equipped with a NanoQuant Plate. Subsequently, 500 ng of the obtained RNA was transcribed using the High-Capacity cDNA Reverse Transcription Kit (Thermo Fisher, Braunschweig, Germany). Polymerase chain reaction was performed using DreamTaq DNA Polymerase Mastermix (Thermo Fisher, Braunschweig, Germany) with the addition of the template cDNA and the desired primer pair (Table S3). The PCR result was applied to a 1.5–2% TAE agarose gel to which Sybr Safe (Thermo Fisher, Braunschweig, Germany) was added as DNA band staining. Bands were captured at 600 nm in the Odyssey XF imager (LI-COR Biosciences, Bad Homburg v. d. Höhe, Germany) and analyzed using Empiria Studio 2.2. 

### 4.6. Agarose Gel DNA Band Analysis

For sequence analysis of the DNA bands obtained by TAE agarose gel electrophoresis, they were excised from the gel, and the fragments were isolated using the QIAquick Gel Extraction Kit (Qiagen, Hilden, Germany), and subsequent Sanger sequencing was performed (MWG Eurofins, Ebersberg, Germany). Analysis of the obtained sequencing data was carried out using the SnapGene software (GSL Biotech LLC, San Diego, CA, USA).

## 5. Conclusions

Cellular expression models for FD and other lysosomal storage diseases usually use cDNA-based genetic vectors to characterize exonic point mutations, but they cannot decipher splicing effects. Thus, investigations of the splicing mechanism provide insight on the potential damage to an enzyme mutant beyond the amino acid exchange. We established a minigene reporter assay for the investigation of splicing defects of *GLA* gene mutations. Unforeseen splicing malfunction seems to be a common occurrence in Fabry-associated exonic *GLA* gene mutations, even in mutations with a low prediction probability as the case of c.548G>T shows. Mutations far from the direct intron/exon boundary should also be considered. The established assay provides information about abnormal splicing, which may have a detrimental effect on the success of a therapy using pharmacological chaperones. 

## Data Availability

Data are contained within the article and the Supplementary Materials.

## Supplementary Materials

The supporting information can be downloaded at: https://www.mdpi.com/article/10.3390/ijms232315261/s1.
