# Supplementary material for: Abnormal Pre-mRNA Splicing in Exonic Fabry Disease-Causing GLA Mutations
Source: Int J Mol Sci. 2022 Dec 3;23(23):15261. doi: 10.3390/ijms232315261 (PMC9737616; doi:10.3390/ijms232315261)
Supplement: Supplementary file 1 [file ijms-23-15261-s001.zip › Table S1A_B.pdf]

Table S1A. Putative splicing effects of exon mutations at splice sites.

cDNA change	AA change	SNP ID	MaxEnt	HSF	Signal	Interpretation
c.194G>T	p.Ser65Ile	n.d.	-101.5%	13.12%	Broken WT Donor Site	Alteration of the WT Donor site, most probably affecting splicing
c.548G>T	p.Gly183Val	rs398123212	---	---	Alteration of auxiliary sequences	Significant alteration of ESE / ESS motifs ratio (-2)
c.638A>T	p.Lys213Met	rs869312149	---	---	Alteration of auxiliary sequences	Significant alteration of ESE / ESS motifs ratio (-5)
c.638A>G	p.Lys213Arg	rs869312149	---	---	Alteration of auxiliary sequences	Significant alteration of ESE / ESS motifs ratio (-8)

AA = amino acid; n.d. = not determined

Table S1B. Putative splicing effects of exon mutations at cryptic splice sites.

cDNA change	AA change	SNP ID	MaxEnt	HSF	Signal	Interpretation
c.358C>G	p.Leu120Pro	n.d.	-1.75 ⇒ 6.52	55.65 ⇒ 82.79	New Donor splice site	Activation of a cryptic Donor site. Potential alteration of splicing
c.1025G>T	p.Arg342Leu	n.d.	---	---	Alteration of auxiliary sequences	Significant alteration of ESE / ESS motifs ratio (-3)
c.1115T>C	p.Leu372Pro	rs1928127729	4.2 ⇒ 6.23	---	New Donor splice site	Activation of a cryptic Donor site. Potential alteration of splicing

AA = amino acid; n.d. = not determined
