# Supplementary material for: Abnormal Pre-mRNA Splicing in Exonic Fabry Disease-Causing GLA Mutations
Source: Int J Mol Sci. 2022 Dec 3;23(23):15261. doi: 10.3390/ijms232315261 (PMC9737616; doi:10.3390/ijms232315261)
Supplement: Supplementary file 1 [file ijms-23-15261-s001.zip › Table S2.pdf]

Table S2. In vitro enzyme activity of putative splicing *GLA* variants in HEK293H cells.

cDNA change	AA change	AGAL activity [% WT] (former study)		Responder (Yes/No)	AGAL activity [% WT] (2022)		Responder (Yes/No)
		- DGJ	+ DGJ		- DGJ	+ DGJ	
c.194G>T	p.Ser65Ile	0	11.2±1.2	yes	0	11.3±1.2	yes
c.358C>G	p.Leu120Pro	50.1±5.0	62.0±2.6	yes	52.2±4.5	61.6±2.3	yes
c.548G>T	p.Gly183Val	0	6.7±2.1	yes	0	5.8±0.3	yes
c.638A>T	p.Lys213Met	83.4±29.6	82.5±15.5	no	64.6±9.4	66.3±8.9	no
c.638A>G	p.Lys213Arg	68.1±8.5	65.3±11.4	no	50.1±4.4	53.9±4.2	no
c.1025G>T	p.Arg342Leu	0	0	no	0	0	no
c.1115T>C	p.Leu372Pro	0	2.6±0.7	no	0	1.9±0.5	no

AA = amino acid

Positive response to 20 μM DGJ is defined as absolute increase in AGAL ≥5% of wild type (WT) or a relative increase in AGAL activity ≥1.5-fold above baseline plus a minimum of 5% activity (%WT).
