# Supplementary material for: Abnormal Pre-mRNA Splicing in Exonic Fabry Disease-Causing GLA Mutations
Source: Int J Mol Sci. 2022 Dec 3;23(23):15261. doi: 10.3390/ijms232315261 (PMC9737616; doi:10.3390/ijms232315261)
Supplement: Supplementary file 1 [file ijms-23-15261-s001.zip › Table S3.pdf]

Table S3. List of primers used in this study.

Primer ID	Sequence (5´- 3)	Purpose
1F	CCTGCTCATCCTCTGGGAGC	binds rat insulin exon 2 of pDESTsplice (forward)
1R	AGGTCTGAAGGTCACGGGCC	binds rat insulin exon 3 of pDESTsplice (reverse)
2F	GACCCACAAGGTTATAAGCAC	isoform-specific PCR, *GLA* intron 4-5 inclusion (forward)
2R	CAGTTGTGCCACTGCCAGG	isoform-specific PCR, *GLA* intron 4-5 inclusion (reverse)
3F	GACATCCCTGGGGCTAGAG	isoform-specific PCR, *GLA* exon 1 (forward)
3R	GCTCCAGTTGTGCCAATAATT	isoform-specific PCR, *GLA* exon 2 normal splicing (forward)
4R	CTCCAGTTGTGCCACTGGC	isoform-specific PCR, *GLA* exon 2 abnormal splicing (-12 nucleotides) (forward)
GLA_ex1_HindIII	TCCCAAGCTTAGTAATTTATTGGGCGCCTTTGTC	Amplification of human *GLA* promoter, exon 1 and intron 1-2 region (forward)
GLA_ex1_BamHI	TCCCGGATCCCCATCCCAGGAAAGGTCACACAG	Amplification of human *GLA* promoter, exon 1 and intron 1-2 region (reverse)
GLA_ex1(2)_BamHI	TCCCGGATCCTTAGGGCGGGAATATTAACGGGATAAG	Amplification of human *GLA* exon 2 and intron 2-3 region (forward)
GLA_ex1(2)_NotI	TCCCGCGGCCGCAGGGCTGTTTCTTTTTTTCCTTGTTTTTTTTTTG	Amplification of human *GLA* exon 2 and intron 2-3 region (reverse)
c194G>T_fwd	CAGATTCCTGCATCATGTATCAGATATTGGG	Mutagenesis of GLA exon 1 (c.194G>T)
c194G>T_rev	CCCAATATCTGATACATGATGCAGGAATCTG	Mutagenesis of GLA exon 1 (c.194G>T)
