# Supplementary figures and images for: Abnormal Pre-mRNA Splicing in Exonic Fabry Disease-Causing GLA Mutations
Source: Int J Mol Sci. 2022 Dec 3;23(23):15261. doi: 10.3390/ijms232315261 (PMC9737616; doi:10.3390/ijms232315261)

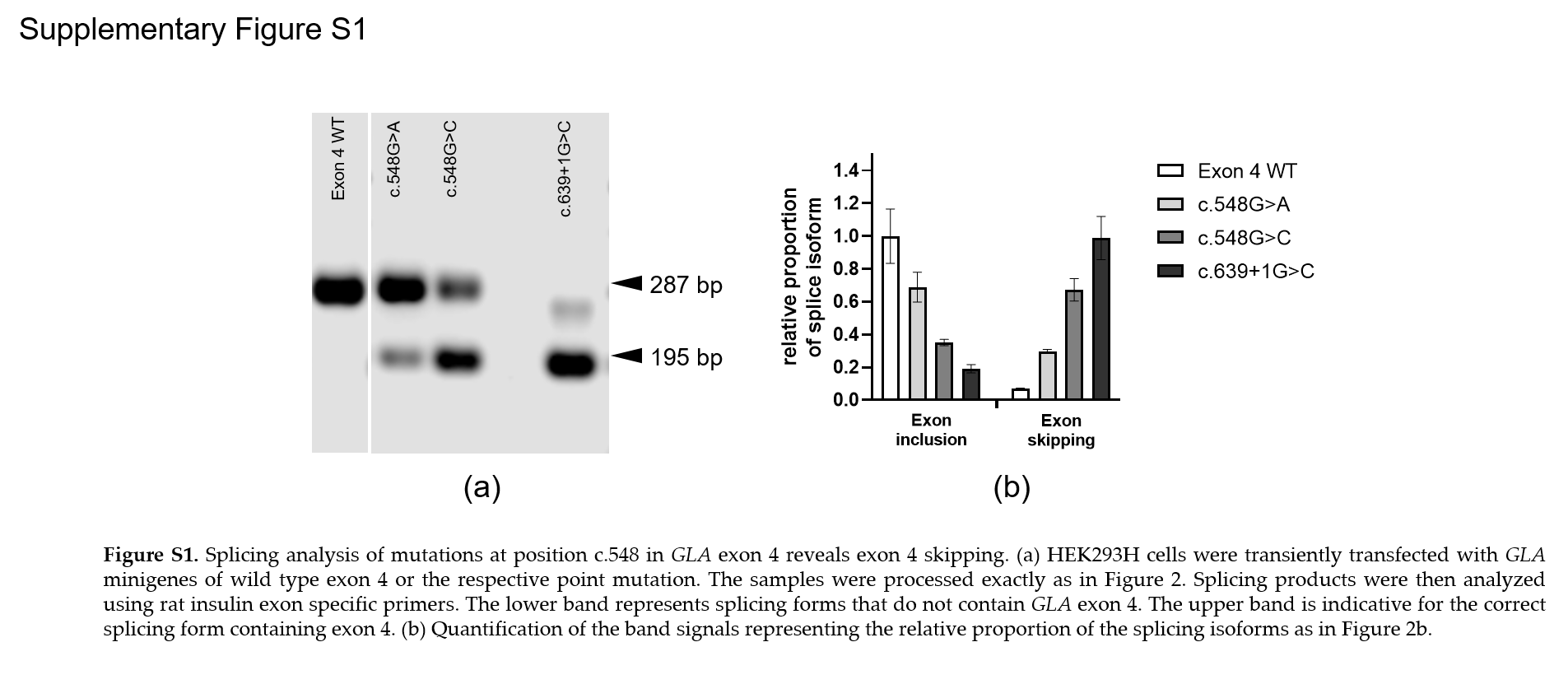

Supplement: Supplementary file 1 [file ijms-23-15261-s001.zip › Suppl. Figure S1.tif]
